# Beyond Nucleotide Excision Repair: The Importance of XPF in Base Excision Repair and Its Impact on Cancer, Inflammation, and Aging

**DOI:** 10.3390/ijms252413616

**Published:** 2024-12-19

**Authors:** Dhara Gohil, Rabindra Roy

**Affiliations:** Department of Oncology, Lombardi Comprehensive Cancer Center, Georgetown University, Washington, DC 20057, USA; dg1061@georgetown.edu

**Keywords:** XPF (ERCC4), nucleotide excision repair (NER), base excision repair (BER), 5′-Gap pathway (5′ Gap LP-BER pathway), RECQ1

## Abstract

DNA repair involves various intricate pathways that work together to maintain genome integrity. XPF (ERCC4) is a structural endonuclease that forms a heterodimer with ERCC1 that is critical in both single-strand break repair (SSBR) and double-strand break repair (DSBR). Although the mechanistic function of ERCC1/XPF has been established in nucleotide excision repair (NER), its role in long-patch base excision repair (BER) has recently been discovered through the 5′-Gap pathway. This study briefly explores the roles of XPF in different pathways to emphasize the importance of XPF in DNA repair. XPF deficiency manifests in various diseases, including cancer, neurodegeneration, and aging-related disorders; it is also associated with conditions such as Xeroderma pigmentosum and fertility issues. By examining the molecular mechanisms and pathological consequences linked to XPF dysfunction, this study aims to elucidate the crucial role of XPF in genomic stability as a repair protein in BER and provide perspectives regarding its potential as a therapeutic target in related diseases.

## 1. Introduction

DNA repair involves many pathways to protect the human genome from various endogenous and exogenous sources of damage. Each pathway has a primary role in repairing specific DNA substrates and may have additional backup roles. Numerous DNA repair proteins are involved in these pathways, which must be expressed at optimal levels for efficient repair.

*XPF* and *ERCC4* were previously assumed to be different genes, but the use of somatic cell hybrids has confirmed that they are identical [1]. For consistency, this paper will only refer to the term XPF moving forward. XPF forms a codependent heterodimer complex with ERCC1 and acts as a structural endonuclease [2,3,4]. The XPF active site is at amino acids 640–720, with three metal-binding and/or catalytic sites at 679/680, 701/702, and 733/734 [5]. It shares a common nuclease motif with proteins within the Mus81 family and an archaeal RNA helicase family [5]. The XPF protein adopts an auto-inhibited conformation which prevents non-specific DNA cleavage; however, the protein’s function is activated when it binds to specific DNA substrates [6].

**Figure 1:**
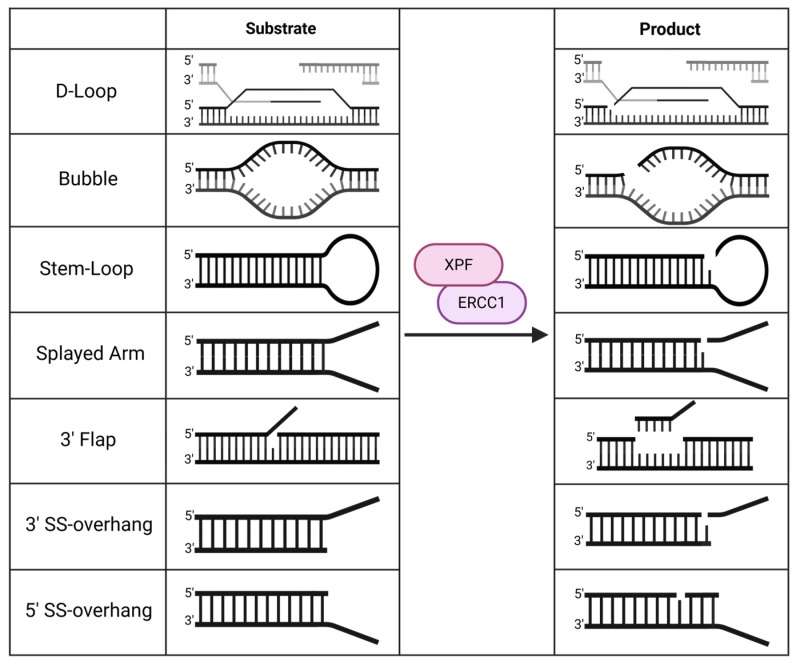
DNA substrates before and after XPF cleavage. Created in https://BioRender.com (accessed on 30 November 2024).

As an endonuclease, XPF cleaves bubble structures, D-loops, or near-DNA junctions in stem-loops/hairpins, splayed arms, flaps, and single-stranded overhanging DNA substrates where there are at least 4–8 unpaired nucleotides [1,7]. The cuts appear to be 15–24 nucleotides away on the 5′ side of the lesion [8,9,10]. This patch size aligns with the optimal single-stranded binding region of RPA, supporting the idea that RPA stimulates the nuclease activity of XPF [11], but the mechanism is not yet known [1]. Both 3′ and 5′ protruding arms can be used to position the protein for its nuclease activity. For splayed arms and flap structures in the 3′ direction, XPF was shown to excise these DNA substrates, while, in duplex DNA, incisions were shown to occur on the 5′ side of the damage in single-stranded DNA [7]. All the DNA substrates before and after XPF cleavage are shown in Figure 1.

In addition to its role in DNA repair, XPF is involved in sister chromatid separation during chromosome segregation [12]. Furthermore, during transcription initiation, XPF may play a role in optimizing the transactivation for genes that can affect cell development [12,13]. Lastly, XPF localizes to the telomeres and interacts with telomere-binding protein 2 (TRF2) [3,14,15]. When TRF2 is inhibited, XPF degrades 3′ G-rich overhangs and causes an increase in chromosomal end fusions; however, XPF overexpression leads to telomere shortening [3,14,15,16]. Through their interactions with SLX4, XPF and TRF2 regulate telomere length.

## 2. Mechanisms of DNA Repair

This section is intended to briefly explain the different repair pathways to highlight the importance of XPF in maintaining genome integrity. Various papers have been cited throughout to provide further sources of information about the roles of the repair proteins mentioned in each pathway.

### 2.1. Single-Strand Break Repair (SSBR)

#### 2.1.1. Nucleotide Excision Repair (NER)

**Figure 2:**
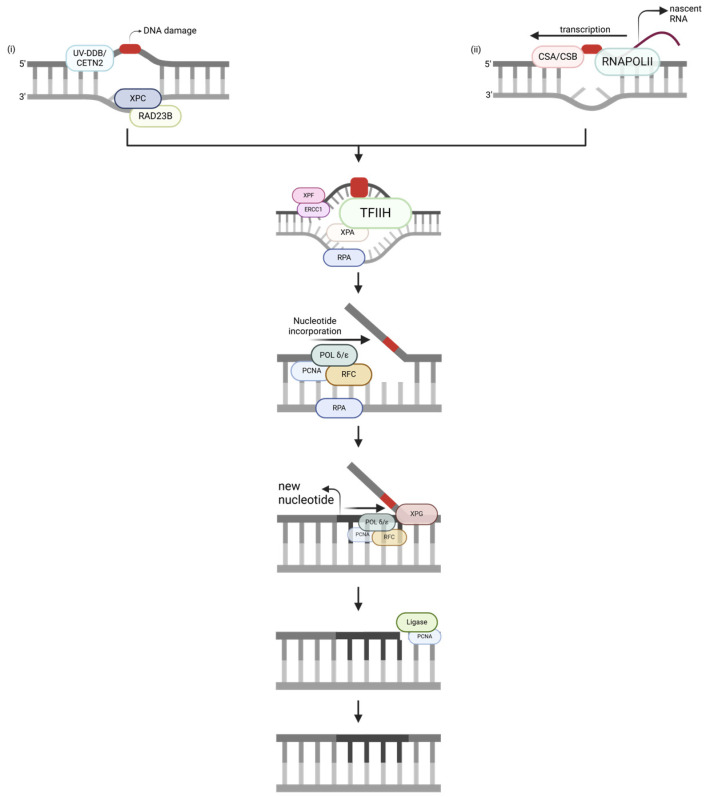
A nucleotide excision repair (NER) pathway, simplified. The strand notation shown for the first step applies to all subsequent steps within the pathway. Unlabeled DNA ends are either polymerase extendable clean 3′-OH groups or 5′-PO_4_ groups that can be ligated for the repair intermediates. (**i**) Global-genome NER (GG-NER). (**ii**) Transcription-coupled NER (TC-NER). Created through https://BioRender.com (accessed on 11 November 2024).

NER (Figure 2) aims to repair bulky DNA adducts formed by various exogenous sources in replicating and non-replicating cells [16]. Global-genome NER (GG-NER) (Figure 2i) repairs damage at a slower rate to protect genome integrity, whereas transcription-coupled NER (TC-NER) (Figure 2ii) is comparatively faster to ensure that transcription occurs correctly [14,17]. GG-NER and TC-NER follow the same pathway—the only difference is the damage recognition process. In GG-NER, UV/DDB and CETN2 work as a complex with XPC and RAD23B to identify the damaged nucleotides [18]. Conversely, for TC-NER, CSA/CSB and stalled RNAPOLII identify the damage [19]. After damage recognition, TFIIH, XPA, and RPA sequentially form a complex. TFIIH contains XPB, which enhances protein binding to DNA, and XPD, which unwinds the DNA to create a bubble structure. Then, XPA joins the complex to verify DNA damage and act as the central scaffolding protein. Afterwards, RPA is bound to ssDNA to protect it from nucleases. The overall stability of this complex allows for the endonucleases ERCC1/XPF and XPG to sequentially act on the damage site, excising a 25–30 nucleotide fragment containing the lesion [12]. The activity of these endonucleases is further enhanced by the presence of RPA [20]. ERCC1/XPF performs the 5′ incision to initiate repair, POLδ/ε incorporates the new nucleotides, and XPG completes the process by making the 3′ incision, allowing ligase to seal the nick [21]. Finally, PCNA ensures the incorporation of the correct number of nucleotides during the repair process.

#### 2.1.2. Base Excision Repair (BER)

**Figure 3:**
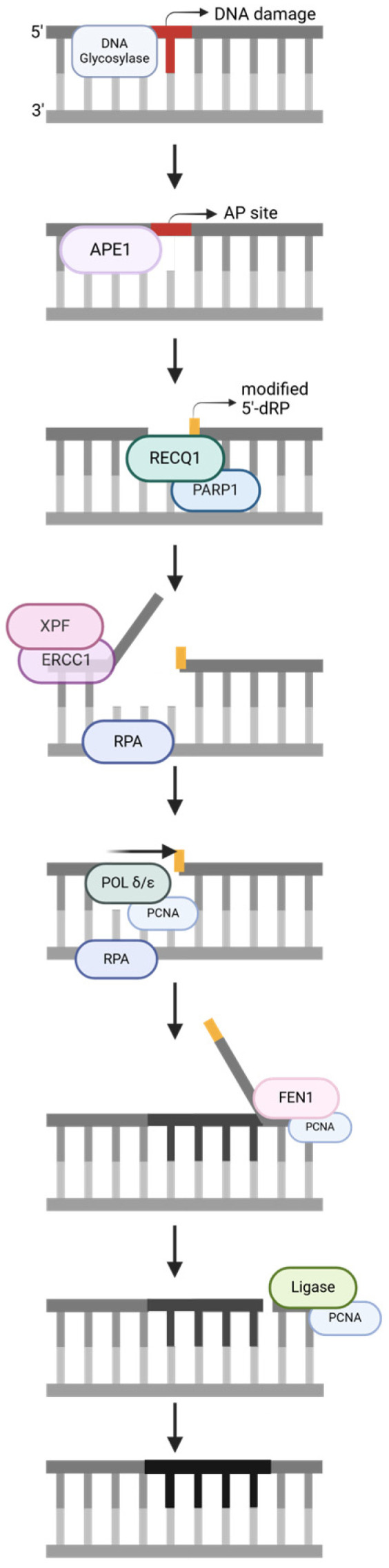
A base excision repair (BER) pathway, simplified. The strand notation shown for the first step applies to all subsequent steps within the pathway. Unlabeled DNA ends are either polymerase extendable clean 3′-OH groups or 5′-PO_4_ groups that can be ligated for the repair intermediates. Created in https://BioRender.com (accessed on 11 November 2024).

BER (Figure 3) aims to repair base lesions that appear to have a minor impact on the helix. Within the various LP-BER sub-pathways, only the 5′-Gap pathway involves ERCC1/XPF [22]. Repair starts with a DNA glycosylase excising the DNA lesion and forming an abasic site (AP site). APE1/PARP1 interact to form 5′-deoxyribose phosphate (5′-dRP), and the cellular environment causes dRP modification. RECQ1 unwinds the helix, and this creates a flap on the 5′ side of the lesion. Then, ERCC1/XPF cleaves the flap, resulting in a nine-nucleotide gap. From there, POLδ/ε fills the gap, FEN1 cleaves the displaced strand, and ligase seals the nick. From the unwinding step onwards, RPA and PCNA are present, localizing the proteins and enhancing their activity [22].

Prior to the discovery of the 5′-Gap pathway, the role of ERCC1/XPF in BER was suggested. Rad1/Rad10, the yeast analog to ERCC1/XPF, can cleave 3′-blocked ends, including 3′-phosphoglycolate (3′-PG) [23]. XPF-deficient CHO cells exposed to hydrogen peroxide showed increased levels of 3′-PG ends [24], while ERCC1/XPF removed a fragment of DNA containing 3′-PG ends that was between five and seven nucleotides from the damaged end [23]. Therefore, ERCC1/XPF is not just a backup for AP endonuclease in processing ROS-induced DNA damage [15] but is also required to trim 3′-blocked ends in mammalian cells [24]. The clonogenic survival assays showed that XPF-deficient cells are more sensitive to ROS-generating agents at a statistically significant rate than the wild-type cells; however, the incision rate was relatively low compared to the amount of XPF tested in vitro [24]. This result supports the need for XPF in BER, but the rate of repair is not efficient. Since XPF typically needs 4–8 unpaired nucleotides before cleaving, the 5′-Gap pathway highlights the need for a 3′-5′ DNA helicase, such as RECQ1, because unwinding the DNA would create a more stable structure for XPF to cleave the flap. Further experimentation is required to determine the proposed increased efficiency of the repair.

PARP1’s impact on DNA repair is dependent on its expression levels: a low amount of PARylation can help relax chromatin and recruit downstream proteins, but excessive amounts can inhibit repair because toxic PARP1 “trapping” can be created on SSB intermediates. In short-patch BER, XRCC1 forms a complex with POLβ and LIG3 to regulate PARP1 engagement in BER [25], and the 5′-Gap pathway can be an alternative mechanism in long-patch BER to regulate PARP1 toxicity. RECQ1 and PARP1 have been shown to exist in a complex [26]; PARP1 facilitates the recruitment of RECQ1 to the damage site, promoting more efficient repair, and RECQ1 inhibits PARP1 PARylation [22]. Since it is not known whether there is complete inhibition, RECQ1 possibly allows for low amounts of PARylation—enough to allow for downstream protein recruitment and prevent NAD^+^ depletion and cell toxicity. For the 5′-Gap pathway, once RECQ1 unwinds the DNA, PARP1 may help recruit ERCC1/XPF, POLδ/ε, and LIG1, while the presence of RECQ1 regulates PARylation to complete the repair. The incised abasic sites created by APE1 have been shown to be the best candidates for the XRCC1 complex [25]. Since the XRCC1 complex cannot regulate PARP1 engagement for all SSB types, the 5′-Gap pathway could be an alternative for modified 5′-dRPs, 3′-PGs, or 3′-P groups.

### 2.2. Double-Strand Break Repair (DSBR)

#### 2.2.1. Single-Strand Annealing (SSA)

**Figure 4:**
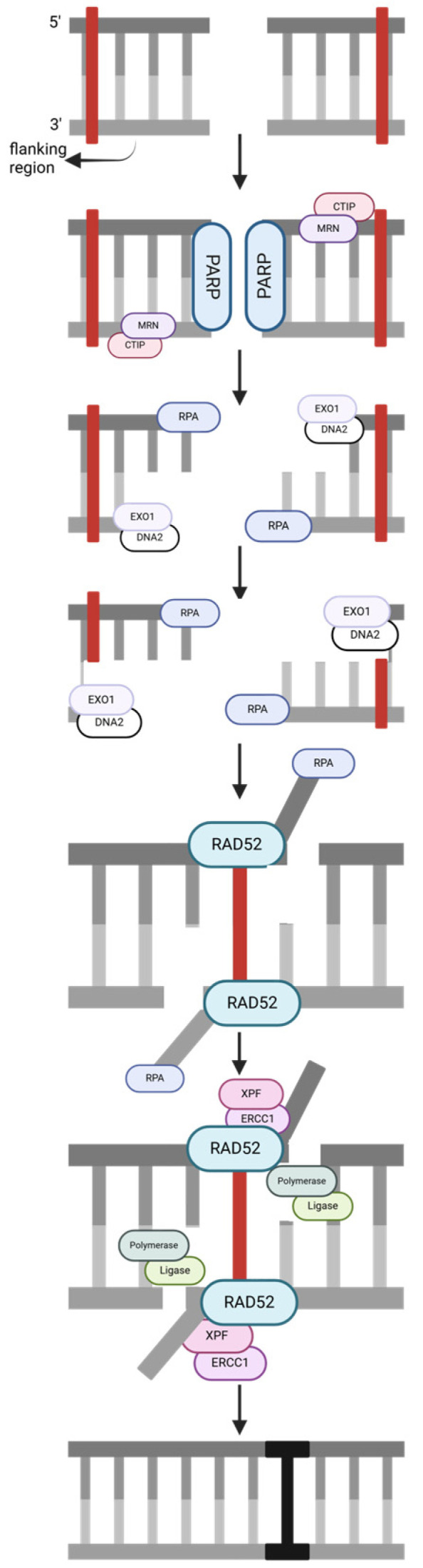
A single-strand annealing (SSA) pathway, simplified. The strand notation shown for the first step applies to all subsequent steps within the pathway. Unlabeled DNA ends are either polymerase extendable clean 3′-OH groups or 5′-PO_4_ groups that can be ligated for the repair intermediates. Created in https://BioRender.com (accessed on 11 November 2024).

Single-strand annealing (SSA) (Figure 4) uses homologous repeats to repair DSBs. Once the flanking region of the strand has been determined, CTIP, MRN, and PARP enter to perform DNA end resection by creating an internal single-strand break at the 5′ strand [27]. Endonucleases EXO1 and DNA2 expose complementary single-strand regions longer than 25 nucleotides, and RPA is present as a binding site. RAD52 and RPA anneal the complementary areas and align the DNA ends, creating non-homologous tails cleaved by ERCC1/XPF. Polymerase and ligase incorporate the new nucleotides and seal the nick.

#### 2.2.2. Homologous Recombination (HR)

**Figure 5:**
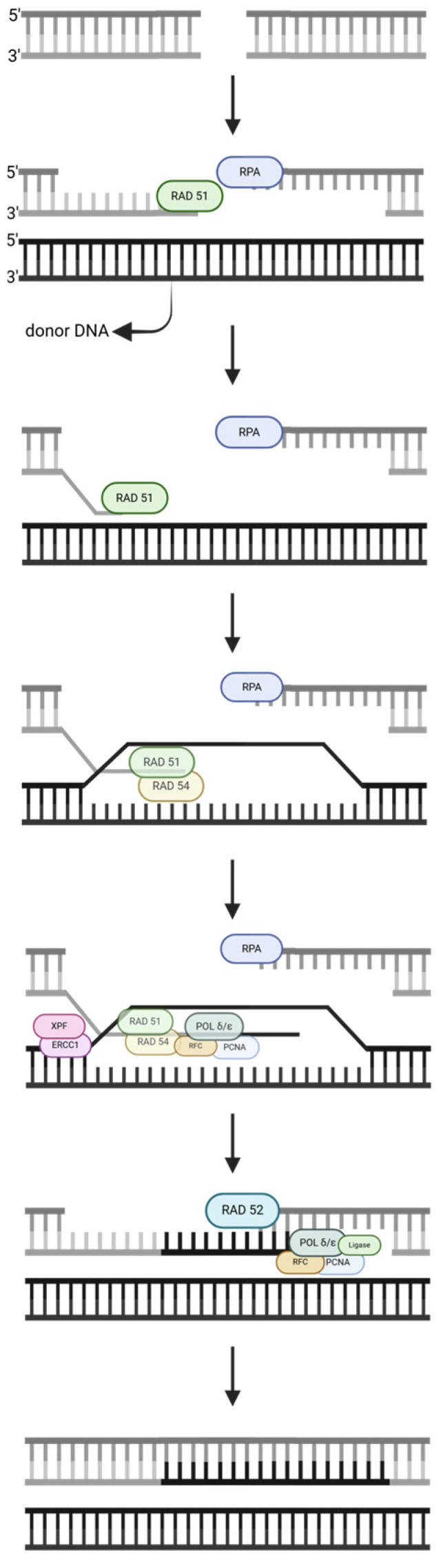
A homologous recombination (HR) pathway, simplified. The strand notation shown for the first step applies to all subsequent steps within the pathway. Unlabeled DNA ends are either polymerase extendable clean 3′-OH groups or 5′-PO_4_ groups that can be ligated for the repair intermediates. Created in https://BioRender.com (accessed on 11 November 2024).

HR (Figure 5) repairs double-strand breaks using a homologous DNA strand as the template strand [28]. RAD51 initiates the homology search and catalyzes the strand exchange [29]. RPA is needed because it binds to ssDNA to prevent the formation of secondary structures and protects DNA from degradation [30]; however, RPA also acts as a competitive inhibitor because it prevents RAD51 from accessing the ssDNA. Mediator proteins are needed to displace RPA inhibition so that RAD51 proteins can form filaments and perform the homology search [28]. Once the homology search is complete, both proteins begin forming the pre-synaptic complex, which involves resection and filament formation. RAD54 joins the protein complex to promote D-loop formation, while PCNA, POLδ/ε, and RFC incorporate new nucleotides. ERCC1/XPF, a component of the resolvase complex, assists to resolve the Holliday junction. RFC helps load PCNA to the damage site, while RAD52 promotes second-strand annealing. The complex (PCNA, POLδ/ε, RFC, and ligase) then returns to continue incorporating new nucleotides until the repair is complete.

### 2.3. Interstrand Crosslink (ICL) Repair

#### 2.3.1. Replication-Independent Repair (RIR)

**Figure 6:**
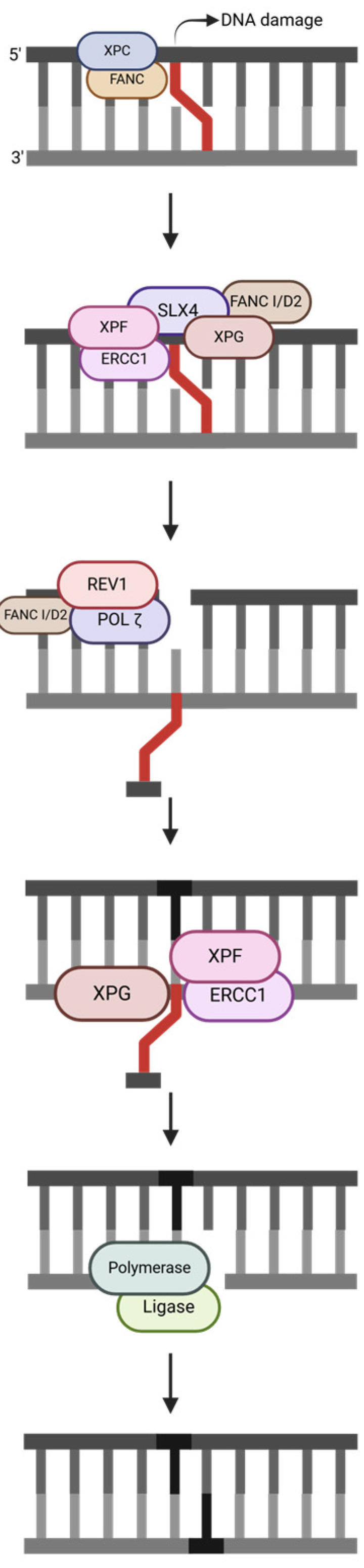
Replication-independent repair (RIR) pathway, simplified. The strand notation shown for the first step applies to all subsequent steps within the pathway. Unlabeled DNA ends are either polymerase extendable clean 3′-OH groups or 5′-PO_4_ groups that can be ligated for the repair intermediates. Created in https://BioRender.com (accessed on 11 November 2024).

RIR (Figure 6) focuses on repairing ICLs in non-dividing cells. XPC initiates repair, and the FANC core is needed to execute FANC I/D2 monoubiquitination for pathway activation [31]. ERCC1/XPF and XPG cleave the lesion, so the damage flips outside the helix and is bound by one strand, while SLX4 is the scaffold protein and FANC I/D2 helps recruit downstream repair proteins. Through TLS, REV1 and POLζ incorporate a single nucleotide [31]. Then, ERCC1/XPF and XPG cleave the lesion on the opposite strand, and the repair is completed by polymerase and ligase.

#### 2.3.2. Replication-Dependent Repair (RDR)

**Figure 7:**
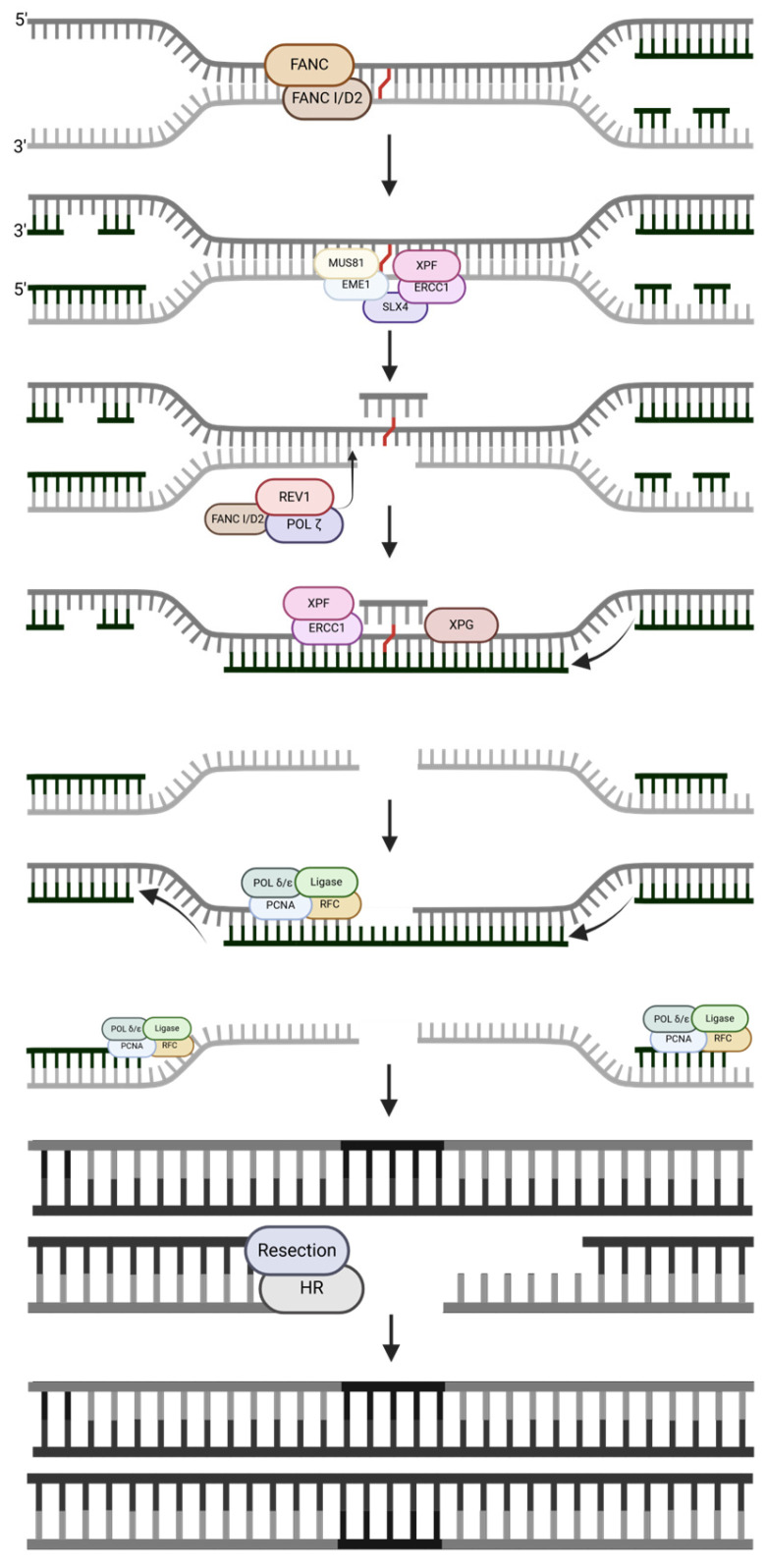
A replication-dependent repair (RDR) pathway, simplified. The strand notation shown for the first step applies to all subsequent steps within the pathway. Unlabeled DNA ends are either polymerase extendable clean 3′-OH groups or 5′-PO_4_ groups that can be ligated for the repair intermediates. Created in https://BioRender.com (accessed on 11 November 2024).

RDR (Figure 7) operates similarly to RIR, but in dividing cells. The ICL is covalently bound to both DNA strands, making it unable to unwind during replication. The FANC core proteins detect the stall in DNA replication, and then FANC I/D2 monoubiquitination activates the pathway [31]. SLX4 creates a scaffold for MUS81/EME1 and ERCC1/XPF to cleave the damage so that the damage orients itself outside of the helix and is then bound to only one DNA strand [12]. Through TLS, REV1 and POLζ synthesize DNA across the damage site, and SNMA1 may be present to digest the nucleotides bound to the crosslink away from the duplex [31]. ERCC1/XPF and XPG cleave the ICL, and that site is repaired by polymerase and ligase [12]. At this point, one of the original strands and its complementary strands have been restored, but the second strand and its complement have formed a DSB. After resection, the remainder of the damage is repaired by HR, with the already-repaired sister chromatids serving as the homology template. The final repair product is two pairs of DNA.

## 3. Diseases

### 3.1. Cancer

Individuals with XPF deficiency may experience the following symptoms: bone marrow failure, predisposition to cancer, congenital malformations, hypersensitivity to crosslinking agents, and chromosome fragility [16]. When hematopoietic stem cells (HSCs) are exposed to endogenous DNA damage, such as ROS, hematopoiesis is compromised [30]. This can lead to repair defects, causing bone marrow failure or hematological malignancies [32]. Although hematopoietic abnormalities are not commonly associated with NER genes, XPF deficiency has been linked to Fanconi anemia (FA) [33]. From a DNA repair standpoint, this can be noted by an increase in crosslink damage [34]. The connection between BER and hematopoiesis can be an area of future research because ROS can impact HSCs’ overall lifespan and their ability to self-renew and differentiate.

Mechanistically, XPF has similar roles in ICL repair and the FA pathway, in which XPF can also be referred to as FANCQ [35]. Mice deficient in XPF, but which have SLX4 KO, share similar developmental and degenerative phenotypes [36,37]. XPF interacts with SLX4, but in BER, XPF does not interact with the scaffold protein XRCC1. Research on the structural comparison of SLX4 and XRCC1 would provide insight into any potential interaction between XPF and XRCC1. The ligase involved in the 5′-Gap pathway has not been determined; if LIG3 is involved, there could be a connection between XPF, XRCC1, and LIG3 because XRCC1 binds with LIG3. 

Depending on the type of mutation, either ICL or NER is compromised [38], but individuals with these mutations do not share the same phenotype as Xeroderma pigmentosum (XP) patients [39,40]. Although XPF mutations make up less than 1% of the cases in FA patients, they can lead to leukemia or breast cancer [41].

Oxidative stress has multiple effects on FA patients. Mice exposed to formaldehyde by inhalation showed increased levels of DNA protein crosslinks, decreased GSH, and increased ROS and MDA across various organs at different levels, but no signs of developmental defects or chromosomal aberrations [42]. The site-specific differences could be due to the differences in antioxidative capacities or that specific DNA repair pathways are more prevalent in certain body areas.

Gemcitabine is a chemotherapeutic that arrests tumor growth, promotes apoptosis [43], and it is also correlated with XPF, possibly through BER. During the G1/S-phase boundary, gemcitabine triphosphate competes with dCTP for incorporation into the DNA. Through this competition, gemcitabine may also inhibit ribonucleotide reductase, which limits the amount of free deoxyribonucleotides in the pool for possible incorporation [44,45,46,47]. Once gemcitabine triphosphate is incorporated, only one additional nucleotide can be added, which blocks further DNA synthesis [48]. Notably, this gemcitabine-induced chain termination might be sequence-context-dependent [49,50]. From a mechanistic standpoint, gemcitabine-induced DNA damage at an SSB with an incorporated gemcitabine-modified nucleotide at or near the 3′-end is said to be repaired through a pathway that allows XPF to process the intermediate structure formed by APE [51]. Alternatively, through the 5′-Gap pathway, after APE1 creates the 5′-dRP (and is modified by the cellular environment), RECQ1 unwinds the DNA with the 3′ block, and XPF cleaves the flap structure created on the 5′ side of the lesion.

Furthermore, gemcitabine induces increased LIG1 levels [52]. Although the ligase involved in the 5′-Gap pathway has not been experimentally determined, it is categorized as an LP-BER sub-pathway, which supports the possibility of LIG1 involvement. The loss of RECQ1 function sensitizes cancer cells to gemcitabine [53], and the helicase function of RECQ1 may be needed to repair gemcitabine-induced DNA damage. Even though RECQ1 was studied from a DSB repair perspective [53], this study, in conjunction with an XPF study regarding gemcitabine [51], shows that the 5′-Gap pathway may be the mechanism of repair for this type of damage, but this needs to be tested experimentally.

### 3.2. Neurodegeneration and Aging

Through patient studies, progressive cognitive impairment was connected to genetic polymorphisms tied with inflammation, DNA repair, and metabolic pathways [54,55].

Although base damage such as 8-oxodG would most likely not be accumulated in neurons because XPF deficiency is predominately tied to NER, the lesions accumulating in neurons are unknown [56]. Moreover, whether XPF is involved in BER through the 5′-Gap pathway was not considered and could be an area of examination in future studies.

Defective DNA repair can contribute to accelerated aging. For example, global and neuron-specific ERCC1 deficiency in the brain impacts cellular pathology and causes a reduction in synaptic plasticity [56]. Mouse models showed an accumulation of oxidative damage points, such as cyclopruine adducts and 4-HNE-induced lesions [57]. Furthermore, senescent cells in ERCC1-deficient animals were comparable to those in wild-type mice that were two years older [57]. Phenotypically, this translates to premature peripheral sensory and motor neuropathy [16]. ERCC1−/∆ mice experienced the same amount of endogenous damage as the wild-type mice but accumulated it faster due to repair deficiencies [57,58,59]. However, through this model, XPF’s repair function in BER through the accumulation of 8-oxodG was not studied.

The symptoms in Cockayne Syndrome (CS) patients include growth failure, cataracts, vascular abnormalities, and pneumonia (as the most frequent cause of death), while the neurology-related symptoms are neurodegeneration, intellectual disability, and microcephaly [60]. From a DNA repair standpoint, CS patients have been shown to have increased levels of ROS and reduced OGG1 expression, postulating the role BER plays in mitigating this disease [61,62,63,64,65]. Patients with cataracts have increased malondialdehyde levels compared to control patients [66], while XPF deficiency was shown to contribute to symptoms such as microcephaly, radio sensitivity, and developmental decay [67]. Finally, a low-glycemic diet may delay the onset of age-related diseases because DNA damage accumulates over time [68,69,70].

### 3.3. Xeroderma Pigmentosum (XP)

Xeroderma pigmentosum (XP) is a skin disorder with phenotypic markers such as severe skin manifestations, profound photosensitivity, dry skin, unusual pigmentation, and a predisposition to skin cancer [71,72,73]. In addition, XP increases predisposition to neurological diseases (in approximately 20% of cases) such as neurodegeneration, dementia, and microcephaly [74,75]. Furthermore, XP can lead to internal cancers such as malignant brain and spinal cord tumors; lung, uterus, breast, pancreas, stomach, kidney, and testicle cancers; and leukemia [76]. However, there is no clear evidence that supports which XP deficiency causes a particular tumor in XP patients [77]. Skin exposed to sunlight is more prone to XP, linking it to an NER deficiency.

Within XP, XPF mutations are rare, making up approximately 1.6% of known cases [71,78]; however, this is thought to be underdiagnosed because the *XPF* gene is critical for fetal development. It is also generally a milder form of XP; patients tend to show sun sensitivity but few tumors [79]. There is conflicting information about the mutation and its effect on endonuclease activity. Although this mutation does not affect nuclease activity [35], it has also been said that the phenotypic signs are caused by diminished endonuclease activity [80]. XPF mutations can interfere with mRNA processing, which can lead to nonsense-mediated mRNA decay, but they can be treated by antisense oligonucleotides that aim to restore expression and DNA repair activities [71].

### 3.4. Fertility

ERCC1/XPF deficiency has been shown to affect fertility. Mouse models showed that both male and female mice were infertile [81]. ERCC1-deficient mice had increased levels of strand breaks and oxidative damage, such as 8-oxodG, in their testes. Furthermore, the male germ cells were more susceptible to apoptosis. Although DNA damage was proposed to be caused by the lack of the recombination repair of NER functions, another justification could be the role of ERCC1 in BER, as that is the primary pathway for 8-oxodG repair.

A sample population study in China showed that a particular ERCC1 polymorphism presents an increased risk for idiopathic azoospermia [82]. In addition to genome stability, various factors can influence spermatogenesis. Although it was hypothesized that mRNA stability may be connected to this disorder, another possible explanation could be that this polymorphism affects DNA repair capabilities.

In human and *C. elegans* oocytes, XPF functions via GG-NER [83], which suggests an evolutionary need to protect the genome within reproductive gonads. For example, rats exposed to carbon tetrachloride showed decreased body weight, reduced tissue GSH levels, lower serum testosterone, upregulated ERCC1 expression, altered testicular histology, and increased lipid peroxidation [84]. Mechanistically, carbon tetrachloride is metabolized by CYP450 enzymes into carbon trichloride free radicals, which bind to the macromolecules that trigger lipid peroxidation [85].

When A549 cells were treated with hydrogen peroxide (H_2_O_2_), ERCC1 expression was downregulated, and XPF expression was upregulated [86]. In glutathione-depleted cells induced with H_2_O_2_, ERCC1 downregulation was abolished, the upregulation of XPF was further upregulated, and NER activity was restored. Since BER is the main repair pathway for oxidative stress, this supports ERCC1/XPF’s role in the 5′-Gap pathway. The difference in the damaging agent could explain the discrepancy between increased ERCC1 expression in the rats exposed to carbon tetrachloride compared to the decreased expression in A549 cells exposed to hydrogen peroxide.

### 3.5. Lipid Peroxidation

Lipid peroxidation is caused by hydroxy radicals attacking the lipid molecules of the cell membrane. ERCC1 deficiency creates more medium- and long-chain lipid peroxidation products, which induces ROS and cellular senescence and inhibits cell proliferation [87]. Various lipid peroxidation products can be formed, and each feature their own consequences.

For example, 4-Hydroxy 2-nonenal (HNE) is formed when free radicals bind to polyunsaturated fatty acids. HNE can lead to inhibited cell proliferation, induced DNA base damage, strand breaks, error-prone translation DNA synthesis, and cellular senescence [87]. ERCC1-deficient mice showed a reduced rate of DNA ligation. Furthermore, ERCC1 KO cells with increased levels of 4-HNE also showed high levels of protein PARylation and 8-oxodG. When all of the proteins in the 5′-Gap pathway operate at optimal levels, PARP1 is needed for RECQ1 activity, but its PARylation activity is inhibited. Due to the ERCC1 KO, the pathway cannot function as needed, resulting in increased PARylation. The increase in base damage and reduced ligation signifies the potential connection between lipid peroxidation and the 5′-Gap pathway.

Notably, 4-HNE activates the NRF2/KEAP1 pathway, which is inversely related to ERCC1 expression [13,32]. Although it can help resist oxidative stress, NRF2/KEAP1 can also create a microenvironment that supports tumor growth and provides drug resistance [88]; this phenomenon is connected to the reduced DNA repair capabilities associated with the loss of ERCC1 expression.

**Figure 8:**
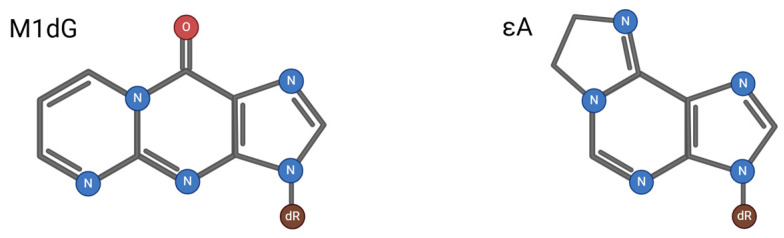
M1dG and εA structures. Created in https://BioRender.com (accessed on 11 November 2024).

Malondialdehyde (MDA) is produced by an increase in free radicals; it is commonly known as a marker of oxidative stress and is a potential threat to transcription [56]. When MDA is bound to DNA, it can form M_1_dG. Although it is known that the primary repair pathway for MDA is NER, BER may play a backup role. In the 5′-Gap pathway, the glycosylase MPG has been shown to repair 1-N^6^-ethanoadenine (εA), which can also be formed during lipid peroxidation [22]. There is a high degree of structural similarity between M_1_dG and εA, as shown in Figure 8. It is plausible that either the same DNA glycosylase or one within the same family may catalyze the removal of M_1_dG from DNA.

### 3.6. Liver and Kidney Dysfunction

Mouse models with ERCC1 mutations showed elevated p53 levels in the liver, brain, and kidney in the perinatal period [35,89]. Knockout caused liver problems, chronic inflammation, no subcutaneous fat, infertility, a short stature, photosensitivity, liver nuclear abnormalities, and death within three weeks [3,68,89,90]. From a DNA repair standpoint, ERCC1 KO affects NER and ICL capabilities [90]. However, mice deficient in NER and ICL repair do not have liver dysfunction, suggesting an additional function for ERCC1/XPF that impacts the liver [91]. Endogenous damage measured in the liver led to increased p21 protein levels [92]; compared to wild-type mice, 8-oxodG levels were nine times higher in ERCC1-deficient mice [92,93]. Although this and premature polyploidy was corrected in the transgene model, increasing the lifespan up to twelve weeks, it caused an ERCC1 deficiency in the kidney, resulting in severe kidney dysfunction, renal failure, and death [93]. Since BER primarily repairs 8-oxodG, the additional function could be the role of ERCC1/XPF in BER.

## 4. Conclusions

XPF is vital to maintaining genomic integrity and cellular function; additionally, as an endonuclease, it is involved in various established pathways, such as nucleotide excision repair, multiple DSBR pathways, and ICL repair. Furthermore, it plays essential roles in sister chromatid separation, transcription initiation, and telomere regulation. The intricate interplay between XPF and other repair proteins highlights its function in a complex network that addresses endogenous and exogenous DNA damage. XPF deficiency increases susceptibility to various diseases, including cancer, neurodegenerative disorders, fertility issues, and skin disorders. Although XPF has only recently been established as a repair protein in BER, it is evident that this pathway is critical for optimal health. Further work needs to be carried out regarding the biological context of the 5′-Gap pathway so that conditions associated with XPF abnormality and/or repair deficiencies are holistically assessed when developing targeted therapies.

## Data Availability

Not applicable.
